# Design, construction and validation of an open-source gold nanoparticle concentration tracking device

**DOI:** 10.1016/j.ohx.2026.e00778

**Published:** 2026-04-26

**Authors:** Sergio Molina-Prados, Vicent Sorribes-Paulo, Lucía Arrufat-Sorli, Heberley Tobón-Maya, Francis Rey-Cortes, Gladys Mínguez-Vega, Carlos Doñate-Buendía

**Affiliations:** Group of Optics-UJI (GROC-UJI), Institute of New Imaging Technologies (INIT), Universitat Jaume I (UJI), 12071 Castellón, Spain

**Keywords:** Arduino, Optical sensor, Nanoparticle monitoring, ESP32-S3, Absorbance

## Abstract

Gold nanoparticles (GNPs) exhibit unique optical properties governed by localized surface plasmon resonance, enabling applications in biomedicine, sensing, catalysis, and photonics. Accurate concentration tracking during fabrication is essential, since particle density strongly affects colloid stability and functional performance. Pulsed laser ablation in liquids (PLAL) has gained recognition as a sustainable route for nanoparticle synthesis, ligand-free, high-purity colloids without chemical by-products. However, monitoring GNP concentration during PLAL typically relies on UV–VIS spectrophotometers, that can be costly, bulky, and difficult to integrate into fabrication workflows. In this work, we present the design, construction, and validation of a compact, open-source alternative for real-time nanoparticle tracking. The device combines a 405 nm laser, a photodiode, and an ESP32-S3 microcontroller for data-acquisition and processing. All design files, firmware, and 3D-printed casing are openly released. Validation experiments show strong agreement with conventional UV–VIS absorption, demonstrating that the proposed device can replace commercial spectrophotometers. By providing a concentration sensor that can be easily integrated in a production chain, this approach reduces the physical footprint and cost of GNPs fabrication facilities, while maintaining reliable monitoring. With a cost below 40 € and modular architecture, the system enables reproducible, accessible, and sustainable monitoring for PLAL and other nanomaterial synthesis processes.

## Graphical abstract

**Figure:**
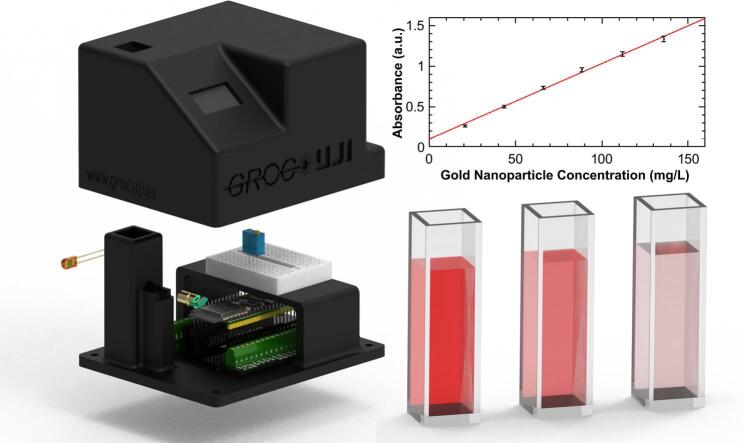

## 1 Specifications table

**Table:** 

Hardware name	NanoGROC-Sens
Subject area	• Engineering and materials science • Chemistry and biochemistry • Educational tools and open-source alternatives to existing infrastructure
Hardware type	• Measuring physical properties and in-lab sensors • Field measurements and sensors • Electrical engineering and computer science
Closest commercial analog	UV–Vis spectroscopy Zuzi, model 4320/3 (3.999,00 €).
Open-source license	CC BY-NC-ND 4.0
Cost of hardware	30.29 €
Source file repository	https://doi.org/10.5281/zenodo.18407862

## 2 Hardware in context

Gold nanoparticles (GNPs) have become a cornerstone in nanoscience due to their unique optical and physicochemical properties. Their localized surface plasmon resonance (LSPR) endows them with remarkable tunability for applications in biomedicine, sensing, catalysis, and photonics [1], [2], [3]. The performance of these applications depends critically on nanoparticle concentration [4], [5], since optical response, stability, and functionalization efficiency are all concentration dependent. However, traditional chemical synthesis methods often suffer from limited reproducibility, reliance on surfactants or stabilizing agents, and the generation of undesirable by-products, which complicate downstream processing and compromise sample purity [6].

As a sustainable alternative, pulsed laser ablation in liquids (PLAL) has emerged as a versatile route for nanoparticle fabrication [7], [8], [9]. This method enables the direct generation of colloids with pristine, ligand-free surfaces [10], while avoiding the chemical residues typical of wet-chemistry approaches. PLAL is inherently flexible, capable of producing nanoparticles of diverse sizes and morphologies by tuning laser and liquid parameter [11] and aligns with green chemistry principles by minimizing waste and hazardous reagents. Such characteristics position PLAL as a competitive and reproducible technique for producing gold colloids with high purity and stability [12], [13], [14].

Despite these advantages, PLAL faces a practical bottleneck: real-time monitoring of nanoparticle concentration during fabrication [15]. High-quality lasers for ablation are increasingly accessible, enabling efficient production, but functional, low-cost spectrometers for in situ concentration tracking remain scarce [16], [17]. Traditionally, UV–Vis spectrophotometers are employed to characterize GNPs, but these instruments are bulky, expensive, and unsuitable for integration into compact fabrication facilities. Yet, concentration monitoring is not merely a diagnostic step, it directly informs production decisions, influencing ablation time, laser parameters, and post-processing requirements [18]. The absence of accessible monitoring tools therefore limits the adoption of PLAL in both research and educational environments.

Some efforts have explored compact or low-cost alternatives to UV–Vis instruments, yet most remain either application-specific or lack open-source availability [19]. Building on these motivations, we propose an open-source device designed for in situ GNP concentration tracking. The system is based on a 405 nm laser diode, a photodiode with sensitivity in the blue region, and an ESP32-S3 microcontroller for acquisition and processing. The design follows open-hardware principles, with 3D-printed casing, accessible electronics, and openly shared firmware, enabling reproducibility and further community-driven improvements. Compared with traditional UV–Vis systems, the proposed device reduces cost, minimizes physical footprint, and allows seamless integration into ablation setups. By reporting its design, validation, and open release, this work contributes a practical tool for concentration monitoring that supports broader adoption of sustainable nanoparticle fabrication.

## 3 Hardware description

**Fig. 1:**
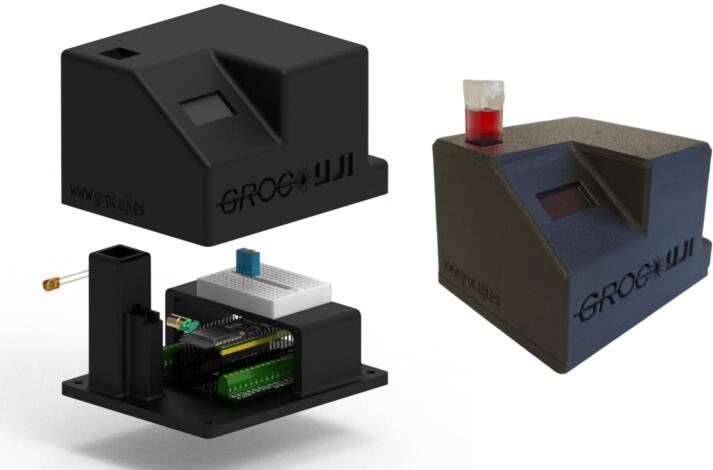
Exploded render of the prototype alongside a photograph of the assembled device.

The proposed device was conceived as a compact, low-cost alternative to conventional spectro-photometers, specifically tailored for the *in-situ* monitoring of gold nanoparticle (GNP) colloids produced by pulsed laser ablation in liquids. Its architecture integrates three essential elements: (i) a 405 nm laser diode module acting as the illumination source, (ii) a silicon photodiode with sensitivity in the blue spectral region to measure transmitted intensity, and (iii) an ESP32-S3 microcontroller responsible for signal acquisition, analog-to-digital conversion, and data management. These components are mounted within a 3D-printed housing that ensures mechanical stability, light-tight operation, and straightforward user access, enabling reproducible measurements in a compact format, see Fig. 1.

Unlike standard UV–Vis spectrophotometers, which are typically benchtop instruments costing several thousand euros, the proposed hardware is deliberately designed to minimize both cost and footprint, with a total assembly cost below 40 €. The reduction in complexity is achieved by constraining the optical path to a single excitation wavelength (405 nm), chosen because it lies away from the surface plasmon resonance (typically around 520 nm, although this value is extremely size-dependent). At 405 nm, the optical absorbance is dominated by the interband transitions of gold, making the measurement directly proportional to the Au concentration and less dependent on particle morphology and size [20], [21]. This target strategy removes the need for broadband lamps, diffraction gratings, or detector arrays, while retaining the essential capability for concentration tracking. Simplification in this way not only lowers costs but also reduces calibration requirements, increases robustness, and facilitates integration into GNP fabrication workflows where continuous operation and compactness are decisive factors.

**Fig. 2:**
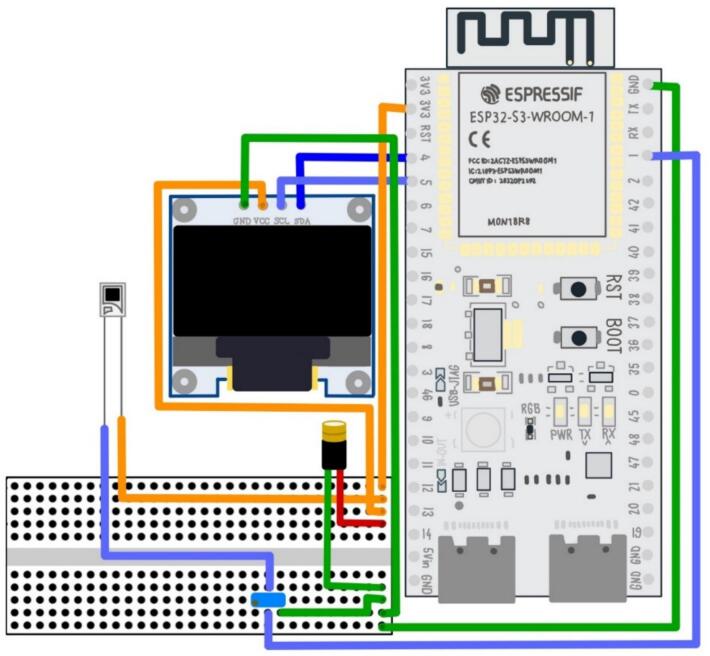
Schematic diagram of the electronic components and their interconnections.

Customization is a central aspect of the design. The modular 3D-printed casing accommodates standard 12.5 × 12.5 mm cuvettes and allows for straightforward alignment and replacement of optical components. Its geometry can be readily adapted to integrate alternative light sources or detectors, permitting the system to be tuned to other plasmonic resonances or absorbance-based assays. The ESP32-S3 microcontroller provides the computational backbone of the system, with a 12-bit ADC for precise digitization and sufficient processing power for real-time signal conditioning. In addition to its low energy consumption and compact form factor, the ESP32-S3 offers advanced functionalities such as Wi-Fi and Bluetooth, opening the possibility for future extensions toward wireless data transfer, cloud integration, or closed-loop control during fabrication, see Fig. 2.

The operational interface of the device is centered on an integrated OLED screen, which displays absorbance readings in real time. A USB Type-C connection establishes communication between the ESP32-S3 and a computer, enabling both data transfer and power supply. For portable operation, the device can also be powered autonomously, without requiring a constant computer link. This flexibility allows the instrument to function either as a standalone unit for rapid characterization or as a continuously logged sensor within larger fabrication setups. Data export can be managed through serial communication, and future firmware adaptations may exploit the wireless features of the microcontroller to further simplify integration into laboratory infrastructures.

Compared to existing low-cost optical sensing platforms, this device directly addresses the requirements of nanoparticle fabrication facilities. Its single-wavelength, transmission-based design is optimized for real-time decision-making during ablation, enabling users to monitor concentration continuously and adjust laser parameters or ablation time accordingly. By eliminating the need for commercial UV–Vis spectrophotometers, the system lowers both the cost and the physical footprint of fabrication setups, reducing barriers for laboratories and educational environments to implement green synthesis strategies with minimal infrastructure investment.

**Fig. 3:**
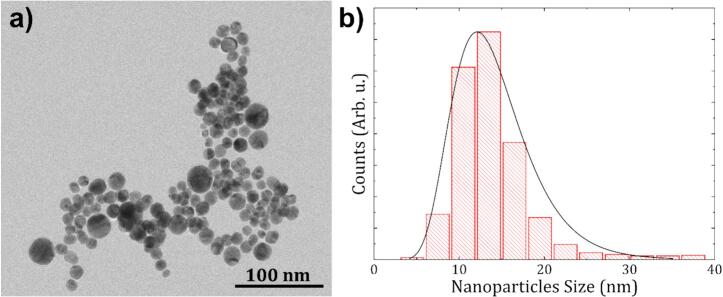
a) TEM image showing spherical Au NPs synthetised by PLAL. b) Histogram of NP diameters fitted with a log-normal distribution, indicating a mean particle size 13.1±0.3 nm.

The Au NPs used to test the NanoGROC-Sens device were synthesized by PLAL technique employing a picosecond laser (Monaco Laser, Coherent) with a collimated beam with a Gaussian waist $\omega_{0}=2mm$ and λ=1034nm wavelength, ablating high-purity gold targets (1 g, 10 mm × 10 mm) submerged in 15 mL of a 10 μM NaCl Milli-Q water solution. The fabrication involved scanning cycles at a speed of 2 m/s focused through a spherical theta lens of 167 mm focal distance. The NPs currently used in this study were analyzed using transmission electron microscopy (TEM) to examine their morphology and size, as shown in Fig. 3 a). Statistical analysis of the resulting colloids, shown in Fig. 3 b), using a Log-Normal distribution fit, revealed an average particle size of 13.1 nm with a standard deviation of 0.3 nm. Furthermore, the analysis confirms that 95% of the NP population is distributed within a narrow range between 5 and 25 nm, ensuring a consistent optical response for the calibration of our device.

In the following sections, we will present how the prototype can be built, calibrated, validated, and tested, providing the necessary details to ensure reproducibility and performance assessment in laboratory conditions.

• **Cost-effective in situ tracking**: Provides an alternative to expensive UV–Vis spectrophotometers, enabling real-time nanoparticle concentration monitoring within compact laboratory setups.
• **Dynamic process optimization:** Facilitates immediate feedback during synthesis, allowing researchers to adjust ablation times or laser parameters to ensure sample productivity or purity.
• **Universal open-source modularity:** Features a 3D-printable and ESP32-S3-powered architecture that can be easily adapted with different light sources or sensors for diverse absorbance-based assays and plasmonic studies.

## 4 Design files summary

**Table:** 

**Design file name**	**File type**	**Open-source license**	**Location of the file**
Base Housing_V4	3D Printing	CC BY-NC-ND 4.0	https://zenodo.org/records/18407862
Cover Housing_V4	3D Printing	CC BY-NC-ND 4.0	https://zenodo.org/records/18407862
Black_Cuvette	3D Printing	CC BY-NC-ND 4.0	https://zenodo.org/records/18407862
NanoGROC_Sens.ino	Software and firmware	CC BY-NC-ND 4.0	https://zenodo.org/records/18407862

• Base Housing_V4, Cover Housing_V4 and Black_Cuvette are device structure files ready for 3D printing.
• NanoGROC_Sens.ino is the software that allows you to calibrate the potentiometer and to measure the GNPs concentration.

## 5 Bill of materials summary

**Table:** 

Designator	Component	Number	Cost per unit −currency	Total cost −currency	Source of materials	Material type
U1	ESP32-S3 DevKitC-1	1	11.38 €	11.38 €	https://goo.su/aZ9pp	−Semi-conductor
J1	ESP32 Adapter	1	2.95 €	2.95 €	https://goo.su/9poo7	−Polymer
D1	OLED Display I2CSSD1306	1	8.49 €	8.49 €	https://goo.su/VSesm	−Semiconductor
R1	Potentiometer 3296 W-1–105	1	3.16 €	3.16 €	https://goo.su/QzgysV	−Composite
D2	SGPD542C8 SiliconPIN Photodiode	1	0.45 €	0.45 €	https://goo.su/CEcxbQa	−Semiconductor
LD1	Violet Laser Diode Module	1	0.99 €	0.99 €	https://goo.su/2rJUq3p	−Semiconductor
BB1	BreadboardMini(Olimex)	1	1.63 €	1.63 €	https://goo.su/lWUfw	−Polymer
C1	Male-to-malejumper wires(Dupont)	5	0.08 €	0.40 €	https://goo.su/iN2SXQf	−Polymer/Metal
C2	Male-to-femalejumper wires	6	0.14 €	0.84 €	https://goo.su/tFEF1	−Polymer/Metal

### 5.1 Build instructions

**Additional items necessary for fabrication and assembly:**

The following tools and materials are required for the fabrication and assembly of the device:

• Soldering iron and solder wire
• Screwdrivers suitable for small electronic components
• M3 screws and nuts
• Cyanoacrylate adhesive
• Double-sided adhesive tape
• Optical diffuser (Scotch® tape, Magic^TM^ Tape, 3 M)
• Computer with the Arduino IDE installed
• USB Type-C to USB Type-C or USB Type-A cable for connection to a computer

**Hardware fabrication – 3D printing:**

The hardware enclosure was fabricated using a Fused Deposition Modelling (FDM) 3D printer (Bambu Lab A1 mini) equipped with a 1.75 mm polylactic acid (PLA) filament inlet. The enclosure was printed using PolyTerra^TM^ Charcoal Black PLA filament (Ø1.75 mm, Batch N° A2412200311), with a nominal density of 1.24 g/cm^3^. Printing parameters were set to an extrusion temperature of 220°C and a heated bed temperature of 60°C. A 0.4 mm stainless steel nozzle and a layer height of 0.2 mm were used to achieve an adequate balance between dimensional accuracy and surface quality.

Printer resolution and dimensional accuracy were carefully considered to ensure correct fitting of all mechanical parts and electronic components, as well as proper alignment of the optical path.

**Mechanical assembly of the enclosure:**

**Fig. 4:**
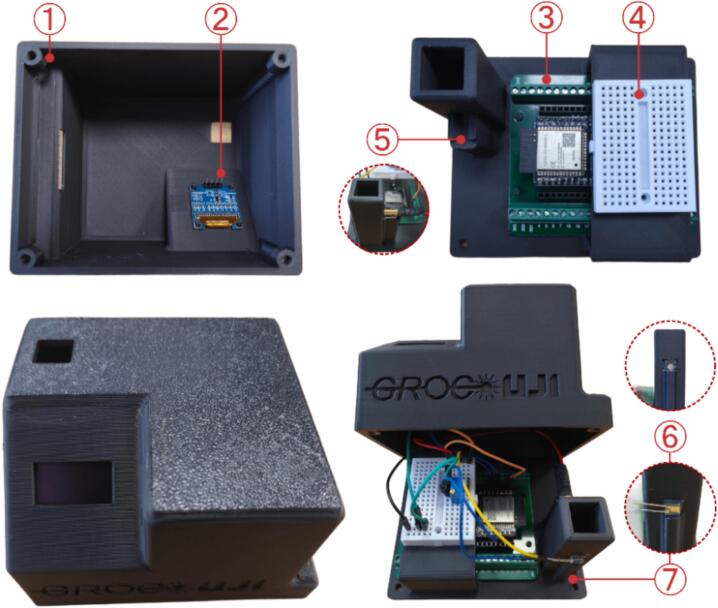
Mechanical assembly instructions of the device, showing the sequential steps required to assemble the enclosure and internal components.

The assembly of the printed enclosure components is straightforward and consists of the following steps, as schematically illustrated in Fig. 4:

1. M3 threaded inserts are installed into the designated recesses on the enclosure cover, enabling secure closure of the device.
2. The OLED display is inserted into the enclosure cover by aligning the display mounting holes with the corresponding printed pillars. The display is retained by a press-fit mechanism and requires no additional fasteners.
3. The ESP32-S3 DevKitC-1 board is mounted onto the base using the integrated printed pillars, which align with the board mounting holes.
4. A small connection board is affixed to the base using double-sided adhesive tape to organize and anchor all electronic connections.
5. The laser diode module is positioned in its designated holder and fixed using a minimal amount of cyanoacrylate adhesive to ensure mechanical stability.
6. A diffuser segment of approximately 5 × 5 mm is cut and adhered to the rear side of the cuvette holder using cyanoacrylate adhesive. This element improves the homogeneous distribution of light across the photodiode active area.
7. The photodiode is mounted laterally onto the dedicated printed base, oriented perpendicular to the laser beam direction.
8. Finally, the enclosure cover is attached to the base, fully enclosing and protecting the system.

**Recommendations to minimize printing defects and ensure proper alignment:**

• Printing supports should be added to all necessary overhanging features to preserve dimensional accuracy.
• As the enclosure consists of two parts, a base housing all electronic components and a cover supporting the OLED display, it is recommended to print the cover upside down to reduce warping and improve surface finish.
• To ensure accurate optical alignment, the laser diode and photodiode should be glued while the laser is powered on. This allows real-time centering of the laser beam on the photodiode active area before the adhesive fully cures.
• Post-processing requirements are minimal and limited to removing printing supports and gently sanding the affected areas to improve the visual finish.

**Assembly of electronic components:**

The electronic assembly procedure is detailed below:

1. Solder the ESP32-S3 module (U1) to male header pins to allow insertion into the terminal adapter board (J1).
2. Connect the ESP32-S3 module to the breadboard using male-to-male jumper wires (C1), as shown in Fig. 2.
3. Solder the photodiode (D2) to two male-to-female jumper wires (C2) and connect it to the breadboard (BB1), as shown in Fig. 2.
4. Connect the potentiometer (R1) to the breadboard according to Fig. 2.
5. Connect the OLED display (D1) to the breadboard and the ESP32-S3 module using male-to-female jumper wires (C2).
6. Connect the laser diode module (LD1) to the breadboard as shown in Fig. 2.

All electronic components must be properly connected to ensure correct operation. Attention should be paid to verifying the ground (GND) and power supply connections to prevent damage to the components.

**Caution:** Whenever possible, lead-free solder should be used. If lead-containing solder is employed, direct contact should be avoided, and hands must be thoroughly washed after handling. Soldering should be performed in a well-ventilated area or using a fume extractor, as soldering fumes may irritate the respiratory system. The soldering iron presents burn and fire hazards and should therefore be handled with care and stored safely when not in use.

**Configuration of Arduino IDE for ESP32-S3 communication:**

To guarantee the ESP32-S3 can communicate with the computer, the following steps are to be followed. Firstly, install ESP32 support in the Arduino IDE (for Arduino versions 1.8.13 or later). Then select the appropriate board model: Tools > Board > esp32 > ESP32S3 Dev Module. To select the appropriate port: Tools > Port and select the one that appears after connecting the module. Finally, in the Tools menu, ensure that “USB CDC on boot” is set to “Enabled”, so the board can establish a serial connection with the computer automatically upon startup and fix the baud rate to 115200.

Install the following libraries in Arduino IDE: Adafruit GFX Library and Adafruit SSD1306 (both by Adafruit) to control the OLED display.

**System calibration:**

As mentioned in section 2-Hardware description, a variable potentiometer was employed. To maximize sensitivity, said potentiometer must be calibrated. Connect the ESP32-S3 module to a computer via the USB Type-C to USB Type-C/Type-A cable, open NanoGROC_Sens.ino and load it onto the module. Now, shine the laser diode onto the photodiode, turn the metallic screw on the bottom left corner in clockwise or anticlockwise direction using a screwdriver, and read the measurements for intensity output in the serial monitor. To ensure accurate measurements of light intensity, it is important that the photodiode does not reach saturation. For reliable results, calibrate the photodiode so that it operates within its linear response range.

## 6 Operation instructions

The operation of the hardware is straightforward and user-friendly. First and foremost, connect the device to the computer as specified above and launch the NanoGROC_Sens.ino file. Verify and upload the program − this might take a few minutes. To make sure the data is interpreted correctly, the laser will be given 10 min to warm up. This will allow the system to reach a stable state. Open the Serial Monitor. The display on the hardware should show the main menu with 3 options for operation: D – Measure Dark, P – potentiometer calibration, M – measurement mode. These operations can be accessed by entering one of the 3 letters listed above into the input field and pressing enter. After Dark and calibration sequence the measurement mode should show the menu with 4 options for operation: B – Measure Blank, S – Measure Sample, E – export data, R – reset system. These operations can be accessed by entering one of the 4 letters listed above into the input field and pressing enter.

It is important to insert the black cuvette (black piece of PLA, 12.5×12.5×40 mm, printable from Black_Cuvette.stl) and the blank cuvette (either the one containing the reference liquid or the colloid to be characterized) into the designed apertures before beginning the dark, calibration and measurement processes, respectively. The dark measurement will measure the electronic noise of the device to better improve final characterization. The calibration menu will measure the light intensity passing through the reference liquid to maximize the number of counts in the photodiode intensity, by adjusting manually the potentiometer. After dark and calibration, the measurement mode will show the menu to start the process of measurement GNP sample. The blank measurement will measure the light intensity $I_{0}$ reaching the photodiode after passing through the reference liquid (the blank medium, providing the baseline). This measurement will be stored in memory to be used later in the absorbance calculation. The measurement sample equally measures light intensity incident on the photodiode after travelling through the colloid to be characterized, $I_{1}$. Samples are required to be named after each measurement for posterior identification by writing in the input field and pressing enter. GNP concentration of each sample is then calculated and stored in memory with the pertaining label. The exporting menu shows a list of the concentrations and associated errors of all samples labelled with the chosen name in the serial monitor to be copied and exported. The reset menu will erase the memory to start over. It should be considered that the program will ask to measure dark and calibrate again when memory is reset.

**Possible modifications:**

On the software side, up to 1000 measurements for different samples are programmed to be taken, but considering the capacity of the microcontroller, this number can be increased if needed by changing the value of the constant MAX_MEASUREMENTS to the desired number. It is also noted that a threshold intensity is set as a lower limit to avoid registration of unintentional measurements. This can also be changed by redefining the value of THRESHOLD (taking into consideration that the maximum intensity that can be measured is 4095 for the 12-bit ADC).

On the hardware side, the modular architecture of the 3D-printed casing allows the system to be adapted for monitoring other types of colloidal NPs (e.g., silver, copper, or carbon quantum dots). To achieve this, the user simply needs to replace the 405 nm laser diode with an alternative LED or laser diode that matches a wavelength where the new material exhibits a linear absorbance-concentration relationship. Once the light source is swapped, the preliminary calibration and validation protocol must be repeated to determine the new regression parameters for the firmware. Beyond material composition, this adaptable methodology can be equally applied to different NP geometries [22]. Moreover, the open-source nature of the software opens the door to future implementations of machine learning models, which could be trained to predict concentrations and morphological variations in more complex optical environments [17], [23].

## 7 Validation and characterization

After acquisition of the baseline ($I_{0}$) and sample ($I_{1})$ transmitted intensities, the embedded software calculates the absorbance of each sample at 405 nm according to Eq. (1).

(1) $$\mathrm{Ab}s_{405}=-log_{10}\left(\frac{I_{1}}{I_{0}}\right)$$

where $I_{0}$ and $I_{1}$ correspond to the transmitted intensities measured for the blank solvent and the nanoparticle-containing sample, respectively.

The operating wavelength of 405 nm was selected because it lies outside the surface plasmon resonance (SPR) peak of GNPs, which is known to be strongly dependent on particle size and morphology. By working at this wavelength, the measured absorbance is predominantly governed by nanoparticle concentration rather than by size-dependent optical effects, enabling a robust linear relationship between absorbance and concentration to be established.

**Calibration procedure:**

**Fig. 5:**
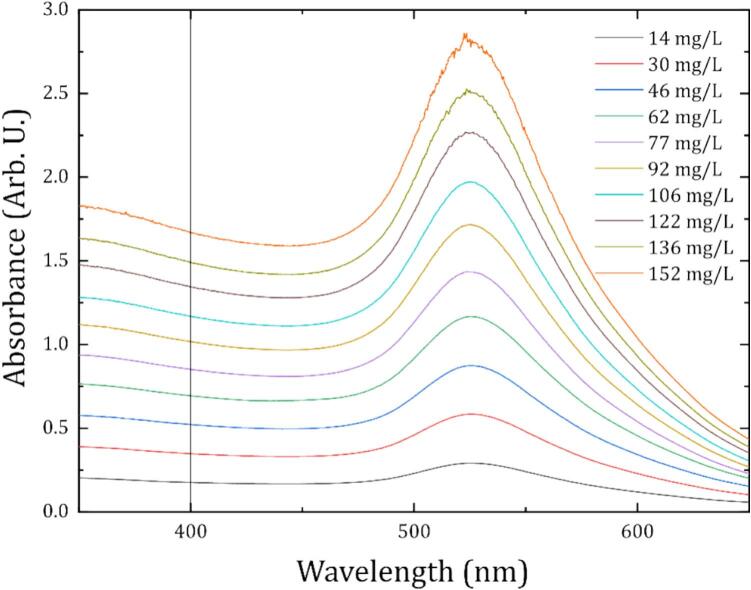
UV–Vis absorption spectra of Au NP colloids. The spectra exhibit the characteristic LSPR peak, indicating the presence of stable, spherical GNPs. The distinct curves correspond to varying colloid concentrations ranging from 14 mg/L to 152 mg/L.

Device calibration was carried out using GNP suspensions of known concentration. These samples were prepared by serial dilution from a stock colloidal solution previously characterised by UV–Vis spectrophotometry using a commercial benchtop spectrometer (Cary Scan 500 UV–Vis-NIR), which served as the reference measurement technique. Fig. 5 illustrates the absorption spectra for varying Au colloid concentrations, ranging from 14 mg/L to 152 mg/L. These samples were prepared by volumetric dilution of a 152 mg/L stock solution (from 10% to 100% v/v). The spectra show a well-defined LSPR peak around 520 nm. These full-spectrum measurements allowed us to validate the absorbance readings at 405 nm, the operational wavelength of our diode laser, confirming a linear relationship between the hardware output and the precise colloid concentration.

**Fig. 6:**
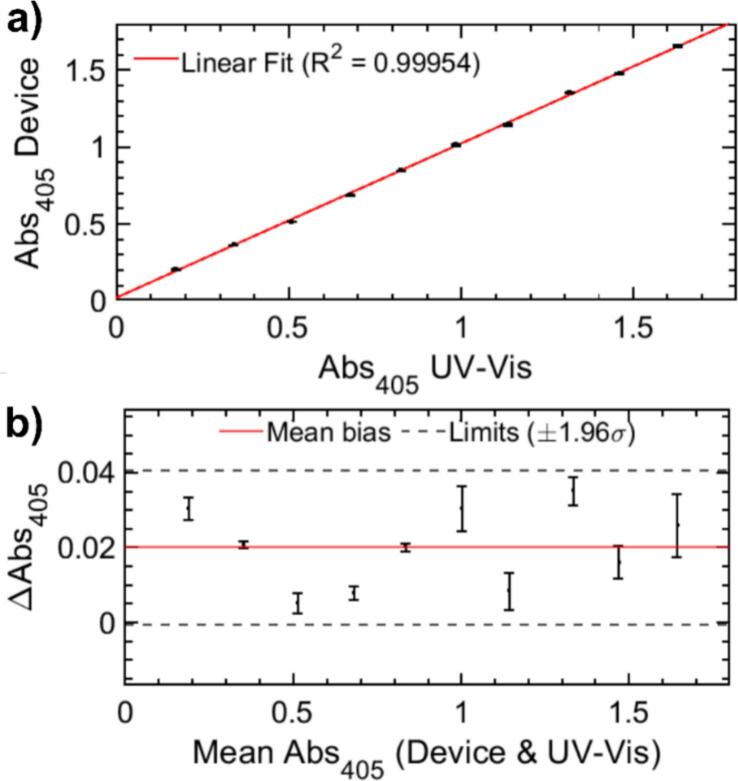
a) Correlation of absorbance measurements at 405 nm between the developed device and a commercial UV–Vis spectrophotometer. b) Bland-Altman plot assessing the agreement between the proposed device and the reference UV–Vis spectrophotometer.

To evaluate the accuracy of the proposed hardware, a cross-validation was performed by comparing the absorbance values recorded by the device ($\mathrm{Ab}s_{405}$ Device) against those obtained with the commercial Cary Scan 500 UV–Vis-NIR spectrophotometer ($\mathrm{Ab}s_{405}$ UV–Vis) for the same set of samples. As shown in Fig. 6 a), the correlation between both instruments is high, following a linear regression y=mx+n with a slope m=0.998±0.007 and an intercept n=0.020±0.005. The determination coefficient of $R^{2}=0.99954$ confirms that the custom-built optical path and the 12-bit ADC processing of the ESP32-S3 provide a performance equivalent to high-end laboratory equipment for single-wavelength measurements at 405 nm.

To assess the agreement between the proposed open-source hardware and the commercial UV–Vis spectrophotometer, comprehensive statistical metrics and a Bland-Altman analysis were employed. The device exhibits an RMSE of 0.0224 and an MAE of 0.0200. The overall average relative error is 3.73%. A Bland-Altman plot, shown in Fig. 6 b) was constructed to evaluate any systematic bias. The analysis reveals a minimal positive mean bias of 0.02 absorbance units, indicating a slight but stable overestimation by the custom device (consistent with the linear intercept). The 95% limits of agreement (±1.96σ) span from −0.0007 to 0.0407, demonstrating that the differences between the low-cost hardware and the reference equipment are statistically minimal and well within the acceptable tolerances for real-time process monitoring.

Final GNP concentrations ranged from approximately 14 mg/L to 152 mg/L. For each sample, absorbance values were recorded using the proposed microcontroller-based system and subsequently correlated with their corresponding concentrations to plot the calibration curve.

**Fig. 7:**
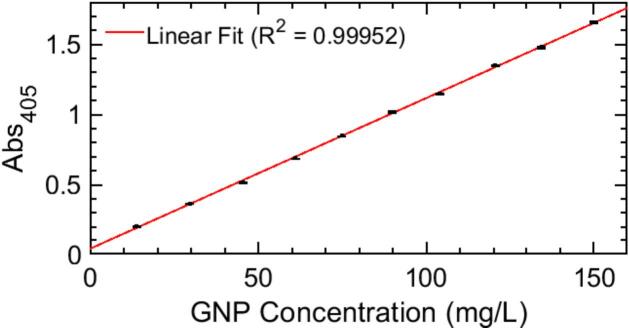
Linear relation between sample absorbance at 405 nm and nanoparticle concentration.

The resulting absorbance-concentration relationship exhibited a linear trend, as shown in Fig. 7. The data were fitted using a linear regression model of the form Eq. (2).

(2) $$\mathrm{Ab}s_{405}=mC+n$$

where C denotes the nanoparticle concentration expressed in mg/L. The fitted parameters yielded values of m=(0.0107±0.0001) and n=(0.041±0.008), confirming the suitability of the device for quantitative concentration measurements within the studied range.

To evaluate the metrological reliability of the proposed device, an intra-sample repeatability study was conducted. A GNP colloid sample (60.7 = mg/l) was measured n = 12 consecutive times. The system yielded a highly consistent response with a SD of 0.20 and a %RSD of 0.33%. This excellent repeatability is attributed to the rigid monolithic design of the 3D-printed housing, which permanently secures the optical axis and prevents user-dependent alignment errors.

**Fig. 8:**
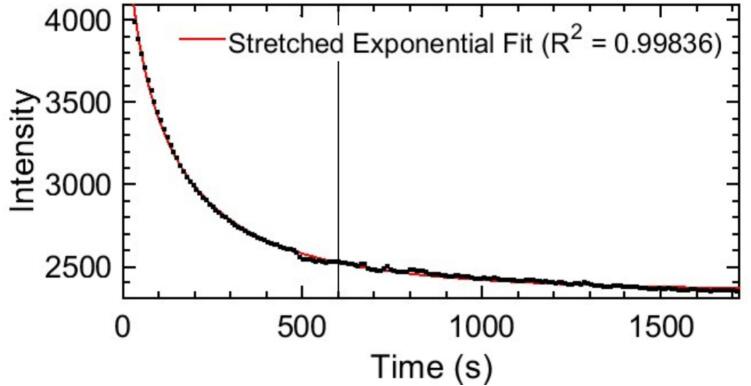
Characterization of the laser diode thermal stability and signal drift. The solid vertical line indicates the optimal stabilization time of t = 10 min, determined to ensure that the signal variation remains below the 2% criterion.

Beyond mechanical stability, to empirically evaluate the thermal effects and signal drift associated with the low-cost laser diode, a continuous time-series stability test was conducted. The raw transmitted intensity of a blank reference was continuously monitored for 30 min from power-on. The system exhibits an initial thermal drift (7% from 10-minute warm-up to minute 30) characterized by a stretched exponential decay $I\left(t\right)=a\cdot exp\left[-{\left(bt\right)}^{c}\right]+d;R^{2}=0.99836$, see Fig. 8. To determine the optimal stabilization period, a kinetic derivative analysis was applied. Given that a standard measurement sequence (Δt) takes 7.5 s, the condition was set that the relative signal variation during this window must remain below 2% (ε=0.02). This tolerance was established as the maximum acceptable error margin to guarantee stable, steady-state conditions during the data acquisition process, in accordance with standard instrumental analysis criteria for maximum permissible source flicker noise and thermal baseline drift characterised in Eq. (3) [24]:

(3) $$\left|I\prime \left(t\right)\cdot \frac{\Delta t}{I\left(t\right)}\right|<0.02$$

The analytical resolution of this condition yields t≈587 s, mathematically justifying the standard 10-minute warm-up period established in the protocol. Furthermore, while macroscopic thermal drift continues to slowly decay over the subsequent hours, it does not impact the analytical reliability of the device. The established protocol inherently compensates for long-term baseline instability by acquiring a fresh blank reference ($I_{0}$) immediately prior to each real-time sample reading.

To further assess inter-day robustness and the impact of the manual calibration process, a re-calibration stress test was performed. The device was subjected to a full reset, including the manual adjustment of the photodiode gain via the potentiometer and the acquisition of a new blank reference ($I_{0}$). After this complete recalibration, the precision metrics remained virtually identical (SD = 0.19, %RSD = 0.31%). This confirms that the manual hardware components do not compromise the instrument's precision. Because the system relies on a relative ratiometric measurement based on the fresh $I_{0}$ blank, any baseline fluctuations are effectively normalized, providing a robust and reproducible analytical tool for continuous monitoring.

The lower and upper limits of validity of the proposed device are determined by distinct physical and instrumental constraints inherent to the optical detection scheme. To formally characterize the sensitivity and analytical limits of the proposed open-source hardware, the LOD and LOQ were calculated based on the SD of the response and the slope of the calibration curve. The SD of the blank (σ=0.0224) was obtained from n = 20 independent measurements of the blank. Applying the standard criteria LOD=3σ/m and LOQ=10σ/m, where m=0.0107 is the slope of the linear fit, the instrument yields an LOD of 6.29 mg/l and an LOQ of 20.96 mg/l. These formal statistical values accurately support and quantify the lower functional threshold of the device, confirming that variations below ∼21mg/l fall outside the reliable quantification limits of the 12-bit ADC architecture. At high GNP concentrations (>150 mg/L), the upper validity limit is governed by optical scattering effects. While the Beer-Lambert law assumes pure absorption, highly concentrated GNP suspensions exhibit significant light scattering in addition to absorption. This effect leads to an overestimation of absorbance and causes deviations from linearity in the absorbance-concentration relationship. Including the most concentrated GNP sample (approximately 170 mg/l and 207 mg/l) resulted in a poor linear regression, characterized by a reduced chi-squared value of $\chi^{2}=3.89$ and a p-value of 0.37%, indicating a statistically inadequate fit. Upon exclusion of this data point, the regression quality improved significantly, yielding a reduced value of $\chi^{2}=1.58$ and a p-value of 19.1%. Experimental evidence supports the interpretation that scattering effects dominate at elevated concentrations.

To rule out optical memory effects, a hysteresis test was performed by evaluating the sample sequence in both ascending and descending concentration orders. The resulting linear regressions showed a negligible variation in the calibration slopes (m = 0.0105 for the ascending sequence versus m = 0.0109 for the descending sequence). This minimal deviation falls within standard experimental dilution errors, confirming that the optoelectronic components do not suffer from hysteresis during standard operation.

Within the validated concentration range, extending up to approximately 150 mg/L, the device demonstrates a stable linear response and yields concentration values in good agreement with reference UV–Vis measurements, with an average relative error of approximately 5%. These results confirm that the proposed system provides accurate and reliable GNP concentration measurements within a well-defined operational window.

## Ethics statements

No human or animal studies were conducted in this work.

## CRediT authorship contribution statement

**Sergio Molina-Prados:** Writing – original draft, Resources, Investigation, Conceptualization. **Vicent Sorribes-Paulo:** Writing – original draft, Validation, Investigation. **Lucía Arrufat-Sorli:** Writing – original draft, Validation, Investigation. **Heberley Tobón-Maya:** Writing – original draft, Methodology. **Francis Rey-Cortes:** Writing – review & editing. **Gladys Mínguez-Vega:** Writing – review & editing, Supervision, Funding acquisition, Conceptualization. **Carlos Doñate-Buendía:** Writing – review & editing, Supervision, Funding acquisition, Conceptualization.

## Declaration of competing interest

The authors declare that they have no known competing financial interests or personal relationships that could have appeared to influence the work reported in this paper.
